# What factors contribute to the meaning of work? A validation of Morin’s Meaning of Work Questionnaire

**DOI:** 10.1186/s41155-020-00167-4

**Published:** 2021-01-04

**Authors:** Anne Pignault, Claude Houssemand

**Affiliations:** 1 Université de Lorraine, Psychology & Neuroscience Laboratory (2LPN, EA7489), 23 boulevard Albert 1er, 54000 Nancy, France; 2 University of Luxembourg, Department of Education and Social Work, Institute for Lifelong Learning & Guidance (LLLG), 2 Avenue de l’Université, L-4365 Esch-sur-Alzette, Luxembourg

**Keywords:** Meaning of work, Structure validation, Psychometrics, Organizational psychology

## Abstract

**Background:**

Considering the recent and current evolution of work and the work context, the meaning of work is becoming an increasingly relevant topic in research in the social sciences and humanities, particularly in psychology. In order to understand and measure what contributes to the meaning of work, Morin constructed a 30-item questionnaire that has become predominant and has repeatedly been used in research in occupational psychology and by practitioners in the field. Nevertheless, it has been validated only in part.

**Method:**

Meaning of work questionnaire was conducted in French with 366 people (51.3% of women; age: (*M* = 39.11, *SD* = 11.25); 99.2% of whom were employed with the remainder retired). Three sets of statistical analyses were run on the data. Exploratory and confirmatory factor analysis were conducted on independent samples.

**Results:**

The questionnaire described a five-factor structure. These dimensions (Success and Recognition at work and of work, α = .90; Usefulness, α = .88; Respect for work, α = .88; Value from and through work, α = .83; Remuneration, α = .85) are all attached to a general second-order latent meaning of work factor (α = .96).

**Conclusions:**

Validation of the scale, and implications for health in the workplace and career counseling practices, are discussed.

## Introduction

Since the end of the 1980s, many studies have been conducted to explore the meaning of work, particularly in psychology (Rosso, Dekas, & Wrzesniewski, 2010). A review of the bibliographical data in PsychInfo shows that between 1974 and 2006, 183 studies addressed this topic (Morin, 2006). This scholarly interest was primarily triggered by Sverko and Vizek-Vidovic’s (1995) article, which identified the approaches and models that have been used and their main results.

Whereas early studies on the meaning of work introduced the concept and its theoretical underpinnings (e.g., Harpaz, 1986; Harpaz & Fu, 2002; Morin, 2003; MOW International Research team, 1987), later research tried to connect this aspect of work with other psychological dimensions or individual perceptions of the work context (e.g., Harpaz & Meshoulam, 2010; Morin, 2008; Morin, Archambault, & Giroux, 2001; Rosso et al., 2010; Wrzesniewski, Dutton, & Debebe, 2003). Nevertheless, scholars, particularly those in organizational and occupational psychology, soon found it difficult to precisely identify the meaning of work because it changes in accordance with the conceptualizations of different researchers, the theoretical models used to describe it, and the tools that are available to measure it for individuals and for groups.

This article first seeks to clarify the concept of the meaning of work (definitions and models) before bringing up certain problems involved in its measurement and the diversity in how the concept has been used. Then the paper focuses on a particular meaning of work measurement tool developed in Canada, which is now widely used in French-speaking countries. At the beginning of the twenty-first century, Morin et al. (2001) developed a 30-item questionnaire to better determine the dimensions that give meaning to a person’s work. The statistical analyses needed to determine the reliability and validity of Morin et al.’s meaning of work questionnaire have never been completed. Indeed, some changes were made to the initial scale, and the analyses only based on homogenous samples of workers in different professional sectors. Thus and even though the meaning of work scale is used quite frequently, both researchers and practitioners have been unsure about whether or not to trust its results. The main objective of the present study was thus to provide a psychometric validation of Morin et al.’s meaning of work scale and to uncover its latent psychological structure.

## Meaning of work: from definition to measurement

### Meaning of work: what is it?

As many scholars have found, the concept of the meaning of work is not easy to define (e.g., Rosso et al., 2010). In terms of theory, it has been defined differently in different academic fields. In psychology, it refers to an individual’s interpretations of his/her actual experiences and interactions at work (Ros, Schwartz, & Surkiss, 1999). From a sociological point of view, it involves assessing meaning in reference to a system of values (Rosso et al., 2010). In this case, its definition depends on cultural or social differences, which make explaining this concept even more complex (e.g., Morse & Weiss, 1955; MOW International Research team, 1987; Steers & Porter, 1979; Sverko & Vizek-Vidovic, 1995).

At a conceptual level, the meaning of work has been defined in three different ways (Morin, 2003). First, it can refer to the meaning of work attached to an individual’s representations of work and the values he/she attributes to that work (Morse & Weiss, 1955; MOW International Research team, 1987). Second, it can refer to a personal preference for work as defined by the intentions that guide personal action (Super & Sverko, 1995). Third, it can be understood as consistency between oneself and one’s work, similar to a balance in one’s personal relationship with work (Morin & Cherré, 2004).

With respect to terms, some differences exist because the meaning of work is considered an individual’s interpretation of what work means or of the role it plays in one’s life (Pratt & Ashforth, 2003). Yet this individual perception is also influenced by the environment and the social context (Wrzesniewski et al., 2003). The psychological literature on the meaning of work has primarily examined its positive aspects, even though work experiences can be negative or neutral. This partiality about the nature of the *meaning of work* in research has led to some confusion in the literature between this concept and that of *meaningful*, which refers to the extent to which work has personal significance (a quantity) and seems to depend on positive elements (Steger, Dik, & Duffy, 2012). A clearer demarcation should be made between these terms in order to specify the exact sense of the meaning of work: “This would reserve ‘meaning’ for instances in which authors are referring to what work signifies (the type of meaning), rather than the amount of significance attached to the work” (Rosso et al., 2010, p. 95).

The original idea of the meaning of work refers to the central importance of work for people, beyond the simple behavioral activity through which it occurs. Drawing on various historical references, certain authors present work as an essential driver of human life; these scholars then seek to understand how work is fundamental (e.g., Morin, 2006; Sverko & Vizek-Vidovic, 1995). The concept of the meaning of work is connected to the centrality of work for the individual and consequently fulfills four different important functions: economic (to earn a living), social (to interact with others), prestige (social position), and psychological (identity and recognition). In this view, the centrality of work is based on an ensemble of personal and social values that differ between individuals as well as between cultures, economic climates, and occupations (England, 1991; England & Harpaz, 1990; Roe & Ester, 1999; Ruiz-Quintanilla & England, 1994; Topalova, 1994; Zanders, 1993).

### Meaning of work: which theoretical model?

The first theoretical model for the meaning of work was based on research in the MOW project (MOW International Research team, 1987), considered the “most empirically rigorous research ever undertaken to understand, both within and between countries, the meanings people attach to their work roles” (Brief, 1991, p. 176). This view suggests that the meaning of work is based on five principal theoretical dimensions: work centrality as a life role, societal norms regarding work, valued work outcomes, importance of work goals, and work-role identification. A series of studies on this theory was conducted in Israel (Harpaz, 1986; Harpaz & Fu, 2002; Harpaz & Meshoulam, 2010), complementing the work of the MOW project (MOW International Research team, 1987). Harpaz (1986) empirically identified six latent factors that represent the meaning of work: work centrality, entitlement norm, obligation norm, economic orientation, interpersonal relations, and expressive orientation.

Another theoretical model on the importance of work in a person’s life was created by Sverko in 1989. This approach takes into account the interactions among certain work values (the importance of these values and the perception of possible achievements through work), which depend on a process of socialization. The ensemble is then moderated by an individual’s personal experiences with work. In the same vein, Rosso et al. (2010) tried to create an exhaustive model of the sources that influence the meaning of work. This model is built around two major dimensions: Self-Others (individual vs. other individuals, groups, collectives, organizations, and higher powers) and Agency-Communion (the drives to differentiate, separate, assert, expand, master, and create vs. the drives to contact, attach, connect, and unite). This theoretical framework describes four major pathways to the meaning of work: individuation (autonomy, competence, and self-esteem), contribution (perceived impact, significance, interconnection, and self-abnegation), self-connection (self-concordance, identity affirmation, and personal engagement), and unification (value systems, social identification, and connectedness).

Lastly, a more recent model (Lips-Wiersma & Wright, 2012) converges with the theory suggested by Rosso et al. (2010) but distinguishes two dimensions: Self-Others versus Being-Doing. This model describes four pathways to meaningful work: developing the inner self, unity with others, service to others, and expressing one’s full potential.

Without claiming to be exhaustive, this brief presentation of the theoretical models of the meaning of work underscores the difficulty in precisely defining this concept, the diversity of possible approaches to identifying its contours, and therefore implicitly addresses the various tools designed to measure it.

### Measuring the meaning of work

Various methodologies have been used to better determine the concept of the meaning of work and to grasp what it involves in practice. The tools examined below have been chosen because of their different methodological approaches.

One of the first kinds of measurements was developed by the international MOW project (MOW International Research team, 1987). In this study, England and Harpaz (1990) and Ruiz-Quintanilla and England (1994) used 14 defining elements to assess agreement on the perception of work of 11 different sample groups questioned between 1989 and 1992. These elements, resulting from the definition of work given by the MOW project and studied by applying multivariate analyses and textual content analyses (*When do you consider an activity as working*? *Choose four statements from the list below which best define when an activity is* “*working,”* MOW International Research team, 1987), can be grouped into four distinct heuristic categories (Table 1).

**Table 1:** Items used to define the concept of work

Burden	Constraint	Responsibility and exchange rationale	Social contributions
b. if someone tells you what to do j. if it is not pleasant m. if you have to do it	a. if you do it in the workplace c. if it is physically strenuous h. if you do it at a certain time (for instance from 8 until 5)	d. if it is one of your tasks g. if it is mentally strenuous k. if you get money for doing it 1. if you have to account for it	e. if you do it to contribute to society f. if, by doing it, you get a feeling of belonging i. if it adds value to something n. if others profit from it

These items were taken from Ruiz-Quintanilla and England (1994). The letter in front of each item corresponds to the initial order of the items (MOW International Research team, 1987)

Similarly, England (1991) studied changes in the meaning of work in the USA between 1982 and 1989. He used four different methodological approaches to the meaning of work: societal norms about work, importance of work goals, work centrality, and definition of work by the labor force. In the wake of these studies, others developed scales to measure the centrality of work in people’s lives, either for the general population (e.g., Warr, 2008) or for specific subpopulations such as unemployed people, on the basis of a rather similar conceptualization of the meaning of work (McKee-Ryan, Song, Wanberg, & Kinicki, 2005; Wanberg, 2012).

Finally, Wrzesniewski, McCauley, Rozin, and Schwartz (1997) developed a rather unusual method for evaluating people’s relationships with their work. Although not directly connected to research on the meaning of work, this study and the questionnaire they used (*University of Pennsylvania Work-Life Questionnaire*) addressed some of the same concepts. Above all, they employed the concepts in a very particular way that combined psychological scales, scenarios, and sociodemographic questions. Through these scenarios (Table 2) and the extent to which the respondents felt like the described characters, their relationship to work was described as either a Job, a Career, or a Calling.

**Table 2:** Scenarios used to measure the relationship to work

Job	Career	Calling
Mr. A works primary to earn enough money to support his life outside of his job. If he was financially secure, he would no longer continue with his current line of work, but would really rather do something else instead. Mr. A’s job is basically a necessity of life, a lot like breathing or sleeping. He often wishes the time would pass more quickly at work. He greatly anticipates weekends and vacations. If Mr. A lived his life over again, he probably would not go into the same line of work. He would not encourage his friends and children to enter his line of work. Mr. A is very eager to retire.	Mr. B basically enjoys his work, but does not expect to be in his current job five years from now. Instead, he plans to move on to a better, higher level job. He has several goals for his future pertaining to the positions he would eventually like to hold. Sometimes his work seems a waste of time, but he knows that he must do sufficiently well in his current position in order to move on. Mr. B can’t wait to get a promotion. For him, a promotion means recognition of his good work, and is a sign of his success in competition with his coworkers.	Mr. C’s work is one of the most important parts of his life. He is very pleased that he is in this line of work. Because what he does for a living is a vital part of who he is, it is one of the first things he tells people about himself. He tends to take his work home with him and on vacations, too. The majority of his friends are from his place of employment, and he belongs to several organizations and clubs relating to his work. Mr. C feels good about his work because he loves it, and because he thinks it makes the world a better place. He would encourage his friends and children to enter his line of work. Mr. C would be pretty upset if he were forced to stop working, and he is not particularly looking forward to retirement.

These scenarios were taken from Wrzesniewski et al. (1997, p. 24)

This presentation of certain tools for measuring the meaning of work reveals a variety of methodological approaches. Nevertheless, whereas certain methods have adopted a rather traditional psychological approach, others are often difficult to use for various reasons such as their psychometrics (e.g., the use of only one item to measure a concept; England, 1991; Wrzesniewski et al., 1997) or for practical reasons (e.g., the participants were asked questions that pertained not only to their individual assessment of work but also to various other parts of their lives; England, 1991; Warr, 2008). This diversity in the possible uses of the meaning of work makes it difficult to select a tool to measure it.

In French-speaking countries (Canada and Europe primarily), the previously mentioned scale created by Morin et al. (2001) has predominated and has repeatedly been used in research in occupational psychology and by practitioners in the field. Nevertheless, there has not been a complete validation of the scale (i.e., different forms of the same tool, only the use of exploratory factor analyses, and no similar structures found) that was the motivation for the current study.

## The present study

The present article conceives of the meaning of work as representing a certain consistency between what an individual wants out of work and the individual’s perception, lived or imagined, of his/her work. It thus corresponds to the third definition of the meaning of work presented above—consistency between oneself and one's work (Morin & Cherré, 2004). This definition is strictly limited to the meaning given to work and the personal significance of this work from the activities that the work implies. Within this conceptual framework, some older studies adopted a slightly different cognitive conception, in which individuals constantly seek a balance between themselves and their environment, and any imbalance triggers a readjustment through which the person attempts to stabilize his/her cognitive state (e.g., Heider, 1946; Osgood & Tannenbaum, 1955). Here, the meaning of work must be considered a means for maintaining psychological harmony despite the destabilizing events that work might involve. In this view, meaning is viewed as an effect or a product of the activity (Brief & Nord, 1990) and not as a permanent or fixed state. It then becomes a result of person-environment fit and falls within the theory of work adjustment (Dawis, Lofquist, & Weiss, 1968).

Within this framework, a series of recurring and interdependent studies should be noted (e.g., Morin, 2003, 2006; Morin & Cherré, 1999, 2004) because they have attempted to measure the coherence that a person finds in the relation between the person’s self and his/her work and thus implicitly the meaning of that work. Therefore, these studies make it possible to understand the meaning of work in greater detail, meaning that it could be used in practice through a self-evaluation questionnaire. The level of coherence is considered the degree of similarity between the characteristics of work that the person attributes meaning to and the characteristics that he/she perceives in his/her present work (Aronsson, Bejerot, & Häremstam, 1999; Morin & Cherré, 2004). Based on semi-structured interviews and on older research related to the quality of life at work (Hackman & Oldham, 1976; Ketchum & Trist, 1992), a model involving 14 characteristics was developed: the usefulness of work, the social contribution of work, rationalization of the tasks, workload, cooperation, salary, the use of skills, learning opportunities, autonomy, responsibilities, rectitude of social and organizational practices, the spirit of service, working conditions, and, finally, recognition and appreciation (Morin, 2006; Morin & Cherré, 1999). Then, based on this model, a 30-item questionnaire was developed to offer more precise descriptions of these dimensions. Table 3 presents the items, which were designed and administered to the participants in French.

**Table 3:** Items from the meaning of work scale by Morin with their theoretical dimensions and exploratory factor analyses

Original theoretical dimensions of the meaning of work 1	Items from the questionnaire with the original item numbers *Work characteristics that have meaning*: (*Caractéristiques du travail qui a du sens*)*:	2	3
Usefulness of work (*Utilité du travail*)	21. Serves some purpose (*Qui sert à quelque chose*)	U	UT
	3. Leads to results that you value (*Qui mène à des résultats que je valorise*)	R	IE
Social contribution (*Contribution sociale*)	9. Is useful to society (*Qui est utile à la société*)	U	UT
	25. Is useful to others (*Qui est utile aux autres*)	U	UT
Rationalization of work (*Rationalité du travail*)	7. Is done efficiently (*Qui est fait de manière efficace*)	A	RT
	2. Its objectives are clear (*Dont les objectifs sont clairs*)	R	RT
	24. Enables you to achieve the goals that you set for yourself (*Qui me permet d’atteindre les objectifs que je me suis fixés)*	R	EFF
Workload (*Charge de travail*)	12. Respects your private life (*Qui respecte ma vie privée*)	S	VP
	18. Workload is adjusted to your capacities (*Dont la charge est ajustée à mes capacités*)	R	RT
Cooperation (*Coopération*)	1. Allows you to have interesting contact with others (*Qui me permet d’avoir des contacts intéressants avec d’autres)*	P	IE
	15. Done in a team spirit (*Qui se fait dans un esprit d’équipe*)	P	ET
Wages (*Salaire*)	23. Gives you wages that provide for your needs (*Qui me donne un salaire qui permet de subvenir à mes besoins*)	S	RT
Using skills (*Exercice des compétences*)	1. Corresponds to your interests and your skills (*Qui correspond à mes intérêts et mes compétences*)	A	EF
	14. You enjoy doing it (*Que j’ai du plaisir à faire*)	P	VP
Occasions for learning (*Occasions d’apprentissage*)	2. Allows you to learn or to improve (*Qui me permet d’apprendre ou de me perfectionner*)	A	EF
	28. Enables you to feel fulfilled (*Qui me permet de m’épanouir*)	P	VP
Autonomy (*Autonomie*)	3. Enables you to use your judgment to solve problems (*Qui permet d’exercer des jugements pour résoudre des problèmes*)	A	IE
	8. Allows you to take initiatives to improve your results (*Qui me permet de prendre des initiatives pour améliorer mes résultats*)	A	EF
	13. You are free to organize things in whatever way you think best (*Que je suis libre d’organiser de la manière qui me semble la plus efficace*)	P	VP
Responsibility (*Responsabilité*)	11. Allows you to have influence over your environment (*Qui me permet d’avoir de l’influence dans mon milieu*)	P	IE
	27. You are responsible (*Dont je suis responsable*)	P	IE
Rectitude of practices (*Rectitude des pratiques*)	4. Is done in an environment in which people are respected (*Qui se fait dans un milieu qui respecte les personnes*)	E	ET
	5. Human values are followed (*Qui respecte les valeurs humaines*)	E	ET
Spirit of service (*Esprit de service*)	22. Gives you the opportunity to serve others (*Qui me donne l’occasion de rendre service aux autres*)	U	UT
	26. You can count on the help of your colleagues when you have problems (*Où je peux compter sur l’aide de mes collègues lorsque j’ai des difficultés*)	S	ET
Health and safety (*Santé et sécurité*)	6. Enables you to consider the future with confidence (*Qui me permet d’envisager l’avenir avec confiance*)	S	RT
	16. Is done in a healthy and safe environment (*Qui se fait dans un environnement sain et sécuritaire*)	S	ET
Recognition (*Reconnaissance*)	17. Your competence is recognized (*Où l’on reconnaît mes compétences*)	R	VP
	19. Your results are recognized (*Où l’on reconnaît mes résultats*)	R	VP
	29. You can count on the support of your superior (*Où je peux compter sur le soutien de mon supérieur*)	R	IE

*P* personal power at work, *U* usefulness of work, *R* success at work, *A* autonomy at work, *S* safety, *E* ethics, *UT* usefulness of work, *VP* personal value, *EF* personal efficacy, *ET* ethics of work, *RT* rationalization of work, *IE* personal influence

(*) = French version. 1 = Morin and Cherré (1999). 2 = Morin et al. (2001) and Morin (2003). 3 = Morin and Cherré (2004)

Some studies for structurally validating this questionnaire have been conducted over the years (e.g., Morin, 2003, 2006, 2008; Morin & Cherré, 2004). However, their results were not very precise or comparable. For example, the number of latent factors in the meaning of work scale structure varied (e.g., six or eight factors: Morin, 2003; six factors: Morin, 2006; Morin & Cherré, 2004), the sample groups were not completely comparable (especially with respect to occupations), and finally, items were added or removed or their phrasing was changed (e.g., 30 and 33 items: Morin, 2003; 30 items: Morin, 2006; 26 items: Morin, 2008). Yet the most prominent methodological problem was that only exploratory analyses (most often a principal component analysis with varimax rotation) had been applied. This scale was entirely relevant from a theoretical point of view because it offered a more specific definition of the meaning of work than other scales and, mainly, because some subdimensions appeared to be linked with anxiety, depression, irritability, cognitive problems, psychological distress, and subjective well-being (Morin et al., 2001). It was also relevant from a practical point of view because it was short and did not take much time to complete. However, its use was questionable because it had never been validated psychometrically, and a consistent latent psychological structure had not been identified across studies.

As an example, two models representing the structure of the 30-item scale are presented in Table 3 (Morin et al., 2001; Morin, 2003, for the first model; Morin & Cherré, 2004, for the second one). This table presents the items, the meaning of work dimensions they are theoretically related to, and the solution from the principal component analysis in each study. These analyses revealed that the empirical and theoretical structures of this tool are not stable and that the latent structure suffers from the insufficient use of statistical methods. In particular, there was an important difference found between the two models in previous studies (Morin et al., 2001; Morin & Cherré, 2004). Only the “usefulness of work” dimension was found to be identical, comprised of the same items in both models. Other dimensions had a maximum of only three items in common. Therefore, it is very difficult to utilize this tool both in practice and diagnostically, and complementary studies must be conducted. Even though there are techniques for replicating explanatory analyses (e.g., Osborne, 2012), such techniques could not be used here because not all the necessary information was given (e.g., all factor loadings, communalities). This is why collecting new data appeared to be the only way to analyze the scale.

More recently, two studies (which applied a new 25-item *meaningful work questionnaire*) were developed on the basis of Morin’s scale (Bendassolli & Borges-Andrade, 2013; Bendassolli, Borges-Andrade, Coelho Alves, & de Lucena Torres, 2015). Even though the concepts of the “meaning of work” and “meaningful work” are close, the two scales are formally and theoretically different and do not evaluate the same construct.

The purpose of the present study was thus to determine the structure of original Morin’s 30-item scale (Morin, 2003; Morin & Cherré, 2004) by using an exploratory approach as well as confirmatory statistical methods (structural equation modeling) and in so doing, to address the lacunae in previous research discussed above. The end goal was thus to identify the structure of the scale statistically so that it can be used empirically in both academic and professional fields. Indeed, as mentioned previously, this scale is of particular interest to researchers because its design is not limited to measuring a general meaning of work for each individual; it can also be used to evaluate discrepancies or a convergence between a person’s own personal meaning of work and a specific work context (e.g., tasks, relations with others, autonomy). Finally, and with respect to previous results, the scale could be a potential predictor of professional well-being and psychological distress at work (Morin et al., 2001).

## Method

### Participants

The questionnaire was conducted with 366 people who were mainly resident in Paris and the surrounding regions in France. The gender distribution was almost equal; 51.3% of the respondents were women. The respondents’ ages ranged from 19 to 76 years (*M* = 39.11, *SD* = 11.25). The large majority of people were employed (99.2%). Twenty percent worked in medical and paramedical fields, 26% in retail and sales, and 17% in human resources (the other respondents worked in education, law, communication, reception, banking, and transportation). Seventy percent had fewer than 10 years of seniority in their current job (*M* = 8.64, *SD* = 9.65). Only three people were retired (0.8%).

### Instrument

Morin’s 30-item meaning of work questionnaire (Morin, 2003; Morin et al., 2001; Morin & Cherré, 2004) along with sociodemographic questions (i.e., sex, age, job activities, and seniority at work) were conducted in French through an online platform. Answers to the meaning of work questionnaire were given on a 5-point Likert scale ranging from 1 (*strongly disagree*) to 5 (*strongly agree*).

### Procedure

Participants were recruited through various professional online social networks. This method does not provide for a true random sample but, owing to it resulting in a potentially larger range of respondents, it enlarges the heterogeneousness of the participants, even if it cannot ensure representativeness (Barberá & Zeitzoff, 2018; Hoblingre Klein, 2018). This point seems important because very homogenous samples were used in previous studies, especially with regard to professions.

Participants were volunteers, and were given the option of being able to stop the survey at any time. They received no compensation and no individual feedback. Participants were informed of these conditions before filling out the questionnaire. Oral and informed consent was obtained from all participants. Moreover, the *Luxembourg Agency for Research Integrity* (LARI on which the researchers in this study depend) specified that according to Code de la santé publique—Article L1123-7, it appears that France does not require research ethics committee [Les Comités de Protection des Personnes (CPP)] approval if the research is non-biomedical, non-interventional, observational, and does not collect personal health information, and thus CNR approval was not required.

Participants had to answer each question in order to submit the questionnaire: If one item was not answered, the respondent was not allowed to proceed to the next question. Thus, the database has no missing data. An introduction presented the subject of the study and its goals and guaranteed the participant’s anonymity. Researchers’ e-mail addresses were given, and participants were informed that they could contact the researchers for more information.

### Data analyses

Three sets of statistical analyses were run on the data:

- Analysis of the items, using traditional true score theory and item response theory, for verifying the psychometric qualities (using mainly R package “psych”). The main objectives of this part of analysis were to better understand the variability of respondents’ answers, to compute the discriminatory power of items, and to verify the distribution of items by using every classical descriptive indicator (mean, standard-deviation, skewness, and kurtosis), corrected item-total correlations, and functions of responses for distributions.
- An exploratory factor analysis (EFA) with an oblimin rotation in order to define the latent structure of the meaning of work questionnaire, performed with the R packages “psych” and “GPArotation”. The structure we retained was based on adequation fits of various solutions (TLI, RMSEA and SRMR, see “List of abbreviations” section at the end of the article), and the use of R package “EFAtools” which helps to determine the adequate number of factors to retain for the EFA solution. Finally, this part of the analysis was concluded using calculations of internal consistency for each factor found in the scale.
- A confirmatory factor analysis using the R package Lavaan and based on the results of the EFA, in order to verify that the latent structure revealed in Step c was valid and relevant for this meaning of work scale. The adequation between data and latent structure was appreciated on the basis of CFI, TLI, RMSEA, and SRMR (see “Abbreviations” section).

For step a, the responses of the complete sample were considered. For steps b and c, 183 subjects were selected randomly for each analysis from the total study sample. Thus, two subsamples comprised of completely different participants were used, one for the EFA in step b and one for the CFA in step c.

Because of the ordinal measurement of the responses and its small number of categories (5-point Likert), none of the items can be normally distributed. This point was verified in step a of the analyses. Thus, the data did not meet the necessary assumptions for applying factor analyses with conventional estimators such as maximum likelihood (Li, 2015; Lubke & Muthén, 2004). Therefore, because the variables were measured on ordinal scales, it was most appropriate to apply the EFA and CFA analyses to the polychoric correlation matrix (Carroll, 1961). Then, to reduce the effects of the specific item distributions of the variables used in the factor analyses, a *minimum residuals* extraction (MINRES; Harman, 1960; Jöreskog, 2003) was used for the EFA, and a *weighted least squares estimator with degrees of freedom adjusted for means and variances* (WLSMV) was used for the CFA as recommended psychometric studies (Li, 2015; Muthén, 1984; Muthén & Kaplan, 1985; Muthén & Muthén, 2010; Yang, Nay, & Hoyle, 2010; Yu, 2002).

The size of samples for the different analyses has been taken into consideration. A model structure analysis with 30 observed variables needs a recommended minimum sample of 100 participants for 6 latent variables, and 200 for 5 latent variables (Soper, 2019). The samples used in the present research corresponded to these a priori calculations.

Finally, according to conventional rules of thumb (Hu & Bentler, 1999; Kline, 2011), acceptable and excellent model fits are indicated by CFI and TLI values greater than .90 and .95, respectively, by RMSEA values smaller than .08 (acceptable) and .06 (excellent), respectively, and SRMR values smaller than .08.

## Results

### Item analyses

The main finding was the limited amount of variability in the answers to each item. Indeed, as Table 4 shows, respondents usually and mainly chose the answers *agree* and *strongly agree*, as indicated by the column of cumulated percentages of these response modalities (%). Thus, for all items, the average answer was higher than 4, except for item 11, the median was 4, and skewness and kurtosis indicators confirmed a systematic skewed on the left leptokurtic distribution. This lack of variability in the participants’ responses and the high average scores indicate nearly unanimous agreement with the propositions made about the meaning of work in the questionnaire.

**Table 4:** Distribution and analysis of the 30 items of the scale

Items from the questionnaire *Work characteristics that have meaning*:	*M*	*SD*	*Med*	%	*Skew*	*Kurt*	*rit*
1. Corresponds to your interests and your skills	4.4	.7	4.0	91.8	− 4.5	22.6	.571
2. Allows you to learn or to improve	4.4	.6	4.0	93.7	− 4.4	20.5	.581
3. Enables you to use your judgment to solve problems	4.0	.9	4.0	75.7	− 2.2	4.9	.432
4. Is done in an environment where people are respected	4.5	.8	4.0	92.9	− 4.1	17.6	.634
5. Human values are respected	4.5	.6	4.0	94.0	− 4.6	21.6	.608
6. Enables you to consider the future with confidence	4.3	.8	4.0	88.5	− 3.8	16.8	.648
7. Is done efficiently	4.3	.7	4.0	89.6	− 3.6	15.5	.665
8. Allows you to take initiatives to improve your results	4.3	.7	4.0	90.2	− 3.2	10.2	.642
9. Is useful to society	4.2	.8	4.0	84.7	− 2.9	9.1	.547
10. Allows you to have interesting contact with others	4.3	.7	4.0	88.8	− 3.4	13.2	.608
11. Allows you to have influence over your environment	3.7	.9	4.0	57.7	− 1.3	1.5	.436
12. Respects your private life	4.3	.9	4.0	85.8	− 3.2	10.6	.516
13. You are free to organize things in the way that you think best	4.2	.8	4.0	83.1	− 2.7	8.4	.498
14. You enjoy doing it	4.5	.7	4.0	94.0	− 5.4	33.9	.579
15. Done in a team spirit	4.2	.8	4.0	82.8	− 2.9	10.7	.559
16. Is done in a healthy and safe environment	4.2	.8	4.0	86.3	− 3.3	11.9	.595
17. Your competence is recognized	4.3	.8	4.0	88.3	− 3.7	15.7	.724
18. Workload is adjusted to your capacities	4.0	.8	4.0	78.1	− 2.5	6.7	.562
19. Your results are recognized	4.2	.8	4.0	84.2	− 2.9	9.1	.657
20. Its objectives are clear	4.2	.8	4.0	85.8	− 3.2	12.0	.603
21. Serves some purpose	4.4	.7	4.0	90.7	− 3.9	19.2	.545
22. Gives you the opportunity to serve others	4.2	.8	4.0	82.8	− 2.8	8.7	.549
23. Gives you wages that provide for your needs	4.4	.7	4.0	91.8	− 4.3	21.0	.548
24. Enables you to achieve the goals you set yourself	4.2	.7	4.0	83.9	− 2.4	5.2	.631
25. Is useful to others	4.2	.8	4.0	85.0	− 2.9	9.3	.560
26. You can count on the help of your colleagues when you have problems	4.2	.8	4.0	82.8	− 3.0	10.6	.584
27. You are responsible	4.2	.8	4.0	84.4	− 3.0	10.3	.562
28. Enables you to feel fulfilled	4.4	.7	4.0	88.0	− 3.3	12.8	.642
29. You can count on the support of your superior	4.1	.9	4.0	81.7	− 2.8	8.2	.557
30. Leads to results that you value	4.1	.8	4.0	77.6	− 2.3	6.6	.542

*M* average of the answers to the item, *SD* standard deviation of the answers to the item, *Med* median, % cumulated percentages of answers 4 (*agree*) and 5 (*strongly agree*) for each item, *skew* skewness, *kurt* kurtosis, *rit* corrected item-total correlations

Table 4 also shows that the items had good discriminatory power, expressed by corrected item-total correlations (calculated with all items) which were above .40 for all items. Finally, item analyses were concluded through the application of item response theory (Excel tools using the eirt add in; Valois, Houssemand, Germain, & Belkacem, 2011) which confirmed, by analyses of item characteristic curves (taking into account that item response theory models are parametric and assume that the item responses distributions follow a logistic function, Rasch, 1980; Streiner, Norman, & Cairney, 2015, p. 297), the psychometric quality of each item and their link to an identical latent dimension. These different results confirmed the interest in keeping all items of the questionnaire in order to measure the work-meaning construct.

### Exploratory analyses of the scale

A five-factor solution was identified. This solution explained 58% of the total variance in the responses of the scale items; the TLI was .885, the RMSEA was .074, and the SRMR was .04. The structure revealed by this analysis was relatively simple (saturation of one main factor for each item; Thurstone, 1947), and the communality of each item was high, except for item 11. The solution we retained presented the best adequation fits and the most conceptual explanation concerning the latent factors. Additionally, the “EFAtools” R package confirmed the appropriateness of the chosen solution. Table 5 shows the EFA results, which described a five-factor structure.

**Table 5:** Loadings and communalities of the 30 items from the meaning of work scale

Items	F1 Success and Recognition	F2 Usefulness	F3 Respect	F4 Value	F5 Remuneration	*h*^2^
19. Your results are recognized	**.83**	− .02	− .05	.06	.08	.75
18. Workload is adjusted to your capacities	**.68**	.05	.14	− .22	.13	.60
17. Your competence is recognized	**.65**	− .06	.09	.21	.12	.71
30. Leads to results that you value	**.65**	.26	.04	.01	− .22	.57
29. You can count on the support of your superior	**.54**	.15	.13	− .07	.06	.49
20. Its objectives are clear	**.46**	.10	.11	.02	.19	.49
24. Enables you to achieve the goals you set yourself	**.48**	.00	.16	.24	.03	.55
11. Allows you to have influence over your environment	**.50**	.10	− .14	.26	− .11	.39
25. Is useful to others	**.31**	.09	.20	− .11	**.32**	.47
27. You are responsible	− .02	**.89**	.03	.00	− .07	.79
22. Gives you the opportunity to serve others	− .03	**.70**	.04	− .08	.21	.58
9. Is useful to society	.06	**.63**	.14	.09	− .11	.58
10. Allows you to have interesting contact with others	.05	**.39**	− .02	.31	.24	.55
21. Serves some purpose	.17	**.53**	.10	− .01	− .02	.46
28. Enables you to feel fulfilled	.07	**.56**	− .04	.22	.14	.56
26. You can count on the help of your colleagues when you have problems	.28	**.32**	− .05	.23	.06	.44
5. Human values are respected	− .01	.06	**.94**	.00	− .02	.92
4. Is done in an environment where people are respected	.05	.01	**.74**	.15	.07	.78
6. Enables you to consider the future with confidence	.23	− .02	**.37**	.14	.28	.59
7. Is done efficiently	.10	.15	**.30**	.20	.25	.58
2. Allows you to learn or to improve	− .09	.15	.08	**.70**	.17	.71
1. Corresponds to your interests and your skills	.12	.03	.27	**.59**	− .10	.60
3. Enables you to use your judgment to solve problems	**.30**	.08	− .10	**.47**	− .04	.43
8. Allows you to take initiatives to improve your results	.27	.06	.11	**.53**	.07	.66
12. Respects your private life	.22	− .01	.27	.01	**.44**	.56
16. Is done in a healthy and safe environment	**.30**	.12	.13	.02	**.42**	.59
13. You are free to organize things in the way that you think best	.04	.09	.03	.18	**.54**	.49
23. Gives you wages that provide for your needs	.31	− .09	.03	.23	**.39**	.50
15. Done in a team spirit	.08	.26	.18	.01	**.39**	.51
14. You enjoy doing it	.06	.21	.10	**.30**	**.32**	.53

EFA with five factors, oblimin rotation. Bold = loading ≥ .30. *h*^2^ = communality

Nevertheless, the correlation matrix for the latent factors obtained by the EFA (see Table 6) suggested the existence of a general second-order meaning of work factor, because the five factors were significantly correlated each with others. This result could be described as the existence of a general meaning of work factor, which alone would explain 44% of the total variance in the responses.

**Table 6:** Correlations between the latent factors from the EFA, Cronbach’s alpha, and McDonald omega for each dimension and general factor

	F1	F2	F3	F4	F5	Alpha	Omega
F1						.90	.93
F2	.46					.88	.92
F3	.48	.57				.88	.91
F4	.46	.42	.34			.83	.85
F5	.44	.29	.48	.34		.85	.87
General						.96	.97

F1: success and recognition at work and from work; F2: usefulness; F3: respect; F4: value from and through work; F5: remuneration; general: total scale

### Internal consistency of latent factors of the scale

The internal consistency of each latent factor, estimated by Cronbach alpha and McDonald omega, was high (above .80) and very high for the entire scale (α = .96 and ω = .97). Thus, for S*uccess and Recognition at work and from work*’*s* factor ω was .93, for *Usefulness*’s factor ω was .92, for *Respect*’s factor ω was .91, for *Value from and through work*’s factor ω was slightly lower and equal to .85, and finally for *Remuneration*’*s* factor for which ω was .87.

### Confirmatory factor analyses of the scale

In order to improve the questionnaire, we applied a CFA to this five-factor model to improve the model fit and refine the latent dimensions of the questionnaire. We used CFA to (a) determine the relevance of this latent five-factor structure and (b) confirm the relevance of a general second-order meaning-of-work factor. Although this procedure might appear redundant at first glance, it enabled us to select a definitive latent structure in which each item represents only one latent factor (simple structure; Thurstone, 1947), whereas the EFA that was computed in the previous step showed that certain items loaded on several factors. The CFA also easily verified the existence of a second-order latent meaning of work factor (the first-order loadings were .894, .920, .873, .892, and .918, respectively). Thus, this CFA was computed to complement the previous analyses by refining the latent model proposed for the questionnaire.

According to conventional rules of thumb (Hu & Bentler, 1999; Kline, 2011), although the RMSEA value for the five-factor model was somewhat too high, the CFI and TLI values were excellent (χ^2^ = 864.72, *df* = 400, RMSEA = .080, CFI = .989, TLI = .988). Table 7 presents the adequation fits for both solutions: a model with 5 first-order factors (as EFA suggests), and a model with 5 first-order factors and 1 second-order factor.

**Table 7:** Solutions of confirmatory factor analyses

Indicators	*χ*^2^	*df*	CFI	TLI	RMSEA	SRMR
Model with 5 first-order factors	837.097	395	.989	.988	.078	.073
Model with 5 first-order factors and 1 second-order factor	864.724	400	.989	.988	.080	.075

*χ*^2^ Chi-square, *df* degrees of freedom, *CFI* comparative fit index, *TLI* Tucker-Lewis Index of factoring reliability, *RMSEA* root mean square error of approximation, *SRMR* standardized root mean square residual

**Fig. 1:**
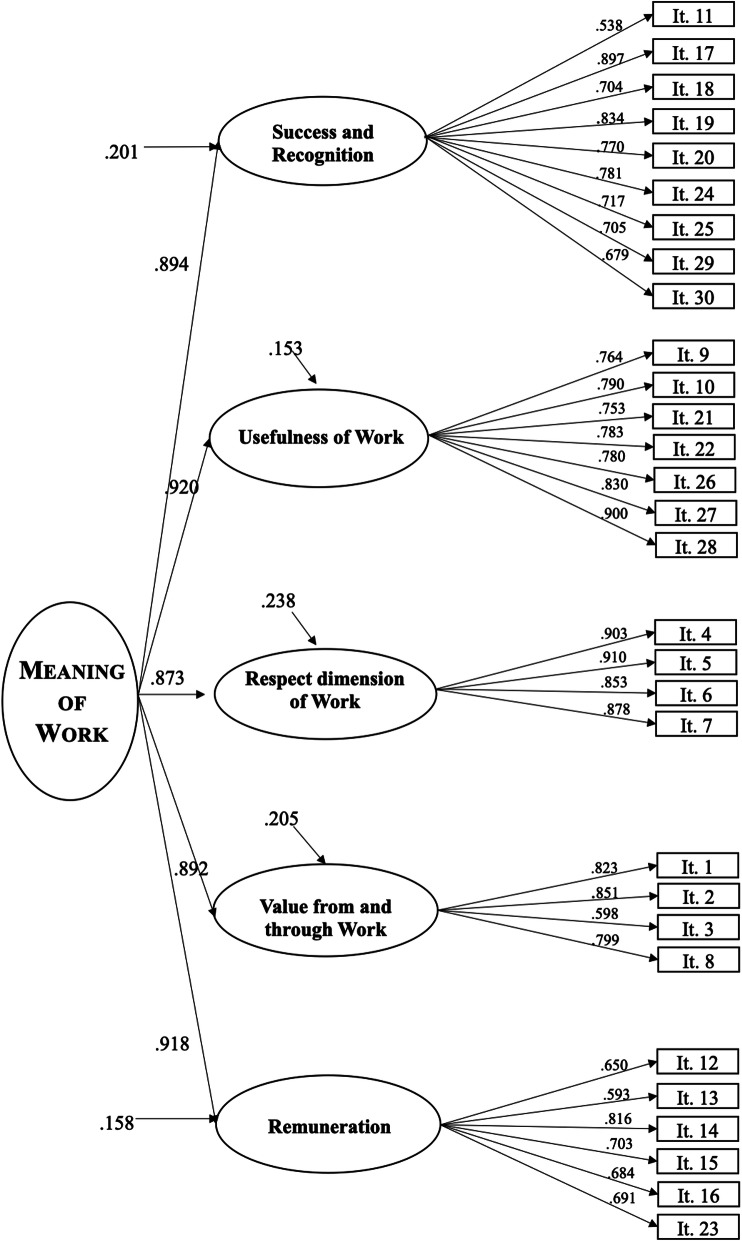
Standardized solution of the structural model of the Meaning of Work Scale

Figure 1 shows the model after the confirmatory test. This analysis confirmed the existence of a simple structure with five factors for the meaning of work scale and with a general, second-order factor of the meaning of work as suggested by the previous EFA.

## Discussion

The objective of this study was to verify the theoretical and psychometric structure of the meaning of work scale developed by Morin in recent years (Morin, 2003; Morin et al., 2001; Morin & Cherré, 2004). This scale has the advantages of being rather short, of proposing a multidimensional structure for the meaning of work, and of making it possible to assess the coherence between the aspects of work that are personally valued and the actual characteristics of the work environment. Thus, it can be used diagnostically or to guide individuals. To establish the structure of this scale, we analyzed deeply the items, and we implemented exploratory and confirmatory factor analyses, which we believe the scale’s authors had not carried out sufficiently. Moreover, we used a broad range of psychometric evaluation methods (traditional true score theory, item response theory, EFA, and structural equation modeling) to test the validity of the scale.

Item analyses confirmed results found in previous studies in which the meaning-of-work scale was administered. The majority of respondents agreed with the proposals of the questionnaire. Thus, this lack of variability is not specific to the present research and its sample (e.g., Morin & Cherré, 2004). Nevertheless, this finding can be explained by different reasons (which could be studied by other research) such as social desirability and the importance of work norms in industrial societies, or a lack of control regarding response bias.

The various versions of the latent structure of the scale proposed by the authors were not confirmed by the statistical analyses seen here. It nevertheless appears that this tool for assessing the meaning of work can describe and measure five different dimensions, all attached to a general factor. The first factor (F1), composed of nine items, is a dimension of recognition and success (e.g., item 17: *work where your skills are recognized*; item 19: *work where your results are recognized*; item 24: *work that enables you to achieve the goals that you set for yourself*). It should thus be named *Success and Recognition at work and from work* and is comparable to dimensions from previous studies (personal success, Morin et al., 2001; social influence, Morin & Cherré, 2004). The second factor (F2), composed of seven items, is a dimension that represents the usefulness of work for an individual, whether that usefulness is social (e.g., Item 22: *work that gives you the opportunity to serve others*) or personal (e.g., Item 28: *work that enables you to be fulfilled*). It can be interpreted in terms of the *Usefulness* of work and generally corresponds to dimensions of the same name in earlier models (Morin, 2003; Morin & Cherré, 2004), although the definition used here is more precise. The third factor (F3), described by four items, refers to the *Respect* dimension of work (e.g., Item 5: *work that respects human values*) and corresponds in part to the factors highlighted in prior studies (respect and rationalization of work, Morin, 2003; Morin & Cherré, 2004). The fourth factor (F4), composed of four items, refers to the personal development dimension and *Value from and through work* (e.g., Item 2: *work that enables you to learn or to improve*). It is in some ways similar to autonomy and effectiveness, described by the authors of the scale (Morin, 2003; Morin & Cherré, 2004). Finally, the fifth and final factor (F5), with six items, highlights the financial and, more important, personal benefits sought or received from work. This includes physical and material safety and the enjoyment of work (e.g., item 14: *work you enjoy doing*). This dimension of *Remuneration* partially converges with the aspects of personal values related to work described in previous research (Morin et al., 2001). Although the structure of the scale highlighted here differed from previous studies, some theoretical elements were nevertheless consistent with each other. To be convinced of this, the Table 8 highlights possible overlaps.

**Table 8:** Final structure the items of the meaning of work scale by Morin and their theoretical dimensions

Final structure of the scale	Items from the questionnaire with the original item numbers *Work characteristics that have meaning*:	1	2
Success and recognition at work and from work	11. Allows you to have influence over your environment	Success at work	Recognition of work
	17. Your competence is recognized		
	18. Workload is adjusted to your capacities 19. Your results are recognized		
	20. Allows you to learn or to improve		
	24. Enables you to achieve the goals that you set for yourself		
	25. Is useful to others		
	29. You can count on the support of your superior		
	30. Leads to results that you value		
Usefulness of work	9. Is useful to society	Usefulness of work Personal power at work	Spirit of service Social contribution
	10. Allows you to have interesting contact with others		
	21. Serves some purpose		
	22. Gives you the opportunity to serve others		
	26. You can count on the help of your colleagues when you have problems		
	27. You are responsible		
	28. Enables you to feel fulfilled		
Respect dimension of work	4. Is done in an environment in which people are respected	Ethics	Rectitude of practices
	5. Human values are followed		
	6. Enables you to consider the future with confidence		
	7. Is done efficiently		
Value from and through work	1. Corresponds to your interests and your skills	Autonomy at work	Mixture
	2. Allows you to learn or to improve		
	3. Enables you to use your judgment to solve problems		
	8. Allows you to take initiatives to improve your results		
Remuneration	12. Respects your private life	Personal power at work Safety	Mixture
	13. You are free to organize things in whatever way you think best		
	14. You enjoy doing it		
	15. Done in a team spirit		
	16. Is done in a healthy and safe environment		
	23. Gives you wages that provide for your needs		

1 = Previous dimensions of Morin et al. (2001) and Morin (2003). 2 = Morin and Cherré (1999)

A second important result of this study is the highlighting of a second-order factor by the statistical analyses carried out. This latent second-level factor refers to the existence of a general meaning of work dimension. This unitary conception of the meaning of work, subdivided into different linked facets, is not in contradiction with the different theories related to this construct. Thus, Ros et al. (1999) defined the meaning of work as a personal interpretation of experiences and interaction at work. This view of meaning of work can confer it a unitary functionality for maintaining psychological harmony, despite the destabilizing events that are often a feature of work. It must be considered as a permanent process of work adjustment or work adaptation. In order to be effective, this adjustment needs to remain consistent and to be globally oriented toward the cognitive balance between the reality of work and the meaning attributed to it. Thus, it has to keep a certain coherence which would explain the unitary conception of the meaning of work.

In addition to the purely statistical results of this study, whereas some partial overlap was found between the structural model in this study and structural models from previous work, this paper provides a much-needed updating and improvement of these dimensions, as we examined several theoretical meaning of work models in order to explain them psychologically. Indeed, the dimensions defined here as *Success and Recognition*, *Usefulness*, *Respect*, *Value*, and *Remuneration* from the meaning of work scale by Morin et al. (2001) have some strong similarities to other theoretical models on the meaning of work, even though the authors of the scale referred to these models only briefly. For example, the dimensions *work centrality as a life role*, *societal norms regarding work*, *valued work outcomes*, *importance of work goals*, and *work-role identification* (MOW International Research team, 1987) concur with the model described in the present study. In the same manner, the model by Rosso et al. (2010) has some similarities to the present structure, and there is a conceptual correspondence between the five dimensions found here and those from their study (*individuation*, *contribution*, *self-connection*, and *unification*). Finally, Baumeister’s (1991), Morin and Cherré’s (2004), and Sommer, Baumeister, and Stillman (2012) studies presented similar findings on the meaning of important life experiences for individuals; they described four essential needs that make such experiences coherent and reasonable (*purpose*, *efficacy*-*control*, *rectitude*, and *self*-*worth*). It is obvious that the parallels noted here were fostered by the conceptual breadth of the dimensions as defined in these models. In future research, much more precise definitions are needed. To do so, it will be essential to continue running analyses to test for construct validity by establishing convergent validity between the dimensions of the various existing meaning of work scales.

It is also interesting to note the proximity between the dimensions described here and those examined in studies on the dimensions that characterize the work context (Pignault & Houssemand, 2016) or in Karasek’s (1979) and Siegrist’s (1996) well-known models, for example, which determined the impact of work on health, stress, and well-being. These studies were able to clearly show how dimensions related to autonomy, support, remuneration, and esteem either contribute to health or harm it. These dimensions, which give meaning to work in a manner that is similar to the dimensions highlighted in the current study (Recognition, Value, and Remuneration in particular), are also involved in health. Thus, it would be interesting to verify the relations between these dimensions and measures of work health.

Thus, the conceptual dimensions of the meaning of work, as defined by Morin (2003) and Morin and Cherré (1999), remained of strong theoretical importance even if, at the empirical level, the scale created on this basis did not correspond exactly. The present study has had the modest merit of showing this interest, and also of proposing a new structure of the facets of this general dimension. One of the major interests of this research can be found in the possible better interpretations that this scale will enable to make. As mentioned above, the Morin’s scale is very frequently used in practice (e.g., in state employment agencies or by Human Resources departments), and the divergent models of previous studies could lead to individual assessments of the meaning of work diverging, depending on the reading grid chosen. Showing that a certain similarity in the structures of the meaning of work exists, and that a general factor of the meaning of work could be considered, the results of the current research can contribute to more precise use of this tool.

At this stage and in conclusion, it may be interesting to consider the reasons for the variations between the structures of the scale highlighted by the different studies. There were obviously the different changes applied to the different versions of the scale, but beyond that, three types of explanation could emerge. At the level of methods, the statistics used by the studies varied greatly, and could explain the variations observed. At the level of the respondents, work remains one of the most important elements of life in our societies. A certain temptation to overvalue its importance and purposes could be at the origin of the broad acceptance of all the proposals of the questionnaire, and the strong interactions between the sub-dimensions. Finally, at the theoretical level, if, as our study showed, a general dimension of meaning of work seems to exist, all the items, all the facets and all the first order factors of the scale, are strongly interrelated at each respective level. As well, small variations in the distribution of responses could lead to variations of the structure.

## Conclusion

The principal contribution of this study is undoubtedly the use of confirmatory methods to test the descriptive models that were based on Morin’s scale (Morin, 2003, 2006; Morin & Cherré, 1999, 2004). The principal results confirm that the great amount of interest in this scale is not without merit and suggest its validity for use in research, both by practitioners (e.g., career counselors and Human Resources departments) and diagnostically. The results show a tool that assesses a general dimension and five subdimensions of the meaning of work with a 30-item questionnaire that has strong psychometric qualities. Conceptual differences from previous exploratory studies were brought to light, even though there were also certain similarities. Thus, the objectives of this study were met.

## Limitations

As with any research, this study also has a certain number of limitations. The first is the sample size used for statistical analyses. Even if the research design respected the general criteria for these kind of analyses (Soper, 2019), it will be necessary to repeat the study with larger samples. The second is the cultural and social character of the meaning of work, which was not addressed in this study because the sample was comprised of people working in France. They can thus be compared with those in Morin’s studies (2003) because of the linguistic proximity (French) of the samples, but differences in the structure of the scale could be due to cultural differences between America and Europe. Nevertheless, other different international populations should be questioned about their conception of the meaning of work in order to measure the impact of cultural and social aspects (England, 1991; England & Harpaz, 1990; Roe & Ester, 1999; Ruiz-Quintanilla & England, 1994; Topalova, 1994; Zanders, 1993). In the same vein, a third limitation involves the homogeneity of the respondents’ answers. Indeed, there was quasi-unanimous agreement with all of the items describing work (see Table 4 and previous results, Morin & Cherré, 2004). It is worth examining whether this lack of variance results from a work norm that is central and promoted in industrialized countries as it might mask broader interindividual differences. Thus, this study’s protocol should be repeated with other samples from different cultures. Finally, a fourth limitation that was mentioned previously involves the validity of the scale. Concerning the content validity and because some items loaded similarly different factors, it could be interesting to verify the wording content of the items, and potentially modify or replace some of them. The purpose of the present study was not to change the content of the scale but to suggest how future studies could analyze this point. Concerning the construct validity, this first phase of validation needs to be followed by other phases that involve tests of convergent validity between the existing meaning of work scales as well as tests of discriminant validity in order to confirm the existence of the meaning of work construct examined here. In such studies, the centrality of work (Warr, 2008; Warr, Cook, & Wall, 1979) should be used to confirm the validity of the meaning of work scale. Other differential, individual, and psychological variables related to work (e.g., performance, motivation, well-being) should also be introduced in order to expand the understanding of whether relations exist between the set of psychological concepts involved in work and individuals’ jobs.

## Data Availability

The datasets generated and/or analyzed during the current study are available from the corresponding author.

## Abbreviations

CFA: Confirmatory factor analyses
CFI: Comparative Fit Index
EFA: Exploratory factor analyses
LARI: Luxembourg Agency for Research Integrity
MOW: Meaning of work
TLI: Tucker Lewis Index of factoring reliability
RMSEA: Root mean square error of approximation
SRMR: Standardized root mean square residual

## References

[CR1] Aronsson G, Bejerot E, Häremstam A (1999). Healthy work: Ideal and reality among public and private employed academics in Sweden. Personal Public Management.

[CR2] Barberá P, Zeitzoff T (2018). The new public address system: why do world leaders adopt social media?. International Studies Quarterly.

[CR3] Baumeister RF (1991). Meaning of life.

[CR4] Bendassolli PF, Borges-Andrade JE (2013). Meaningfulness in work in Brazilian and French creative industries. Spanish Journal of Psychology.

[CR5] Bendassolli PF, Borges-Andrade JE, Coelho Alves JS, de Lucena Torres T (2015). Meaningful work scale in creative industries: A confirmatory factor analysis. Psico-USF.

[CR6] Brief AP (1991). MOW revisited: A brief commentary. European Work and Organizational Psychology.

[CR7] Brief AP, Nord WR (1990). Meaning of occupational work.

[CR8] Carroll JB (1961). The nature of the data, or how to choose a correlation coefficient. Psychometrika.

[CR9] Dawis RV, Lofquist LH, Weiss DJ (1968). A theory of work adjustment (a revision). Minnesota Studies in Vocational Rehabilitation, XXIII.

[CR10] England, G. W. (1991). The meaning of working in USA: Recent changes. *The European Work and Organizational Psychologist*, *1*, 111–124. 10.1111/j.1464-0597.1990.tb01036.x

[CR11] England GW, Harpaz I (1990). How working is defined: National contexts and demographic and organizational role influences. Journal of Organizational Behavior.

[CR12] Hackman JR, Oldham GR (1976). Motivation through the design of work: Test of a theory. Organizational Behavior and Human Performance.

[CR13] Harman HH (1960). Modern Factor Analysis.

[CR14] Harpaz I (1986). The factorial structure of the meaning of work. Human Relations.

[CR15] Harpaz I, Fu X (2002). The structure of the meaning of work: A relative stability amidst change. Human Relations.

[CR16] Harpaz I, Meshoulam I (2010). The meaning of work, employment relations, and strategic human resources management in Israel. Human Resource Management Review.

[CR17] Heider F (1946). Attitudes and cognitive organization. Journal of Psychology.

[CR18] Hoblingre Klein H (2018). Réseaux sociaux professionnels: instruments d’empowerment professionnel ?: Analyse de cas de consultants RH et de recruteurs sur LinkedIn.

[CR19] Hu LT, Bentler PM (1999). Cutoff criteria for fit indexes in covariance structure analysis: Conventional criteria versus new alternatives. Structural Equation Modeling.

[CR20] Jöreskog KG (2003). Factor analysis by MINRES.

[CR21] Karasek RA (1979). Job demands, job decision latitude, and mental strain: Implications for job redesign. Administrative Science Quarterly.

[CR22] Ketchum LD, Trist E (1992). All teams are not created equal. How employee empowerment really works.

[CR23] Kline RB (2011). Principles and practices of structural equation modeling.

[CR24] Li, C. H. (2015). Confirmatory factor analysis with ordinal data: Comparing robust maximum likelihood diagonally weighted least squares. *Behavior Research Method*, 1–14. 10.3758/s13428-015-0619-7. 10.3758/s13428-015-0619-7 26174714

[CR25] Lips-Wiersma M, Wright S (2012). Measuring the meaning of meaningful work: Development and validation of the Comprehensive Meaningful Work Scale (CMWS). Group & Organization Management.

[CR26] Lubke GH, Muthén BO (2004). Applying multigroup confirmatory factor models for continuous outcomes to Likert scale data complicates meaningful group comparisons. Structural Equation Modeling.

[CR27] McKee-Ryan F, Song Z, Wanberg CR, Kinicki AJ (2005). Psychological and physical well-being during unemployment: A meta- analytic study. Journal of Applied Psychology.

[CR28] Morin E, Vandenberghe C, Delobbe N, Karnas G (2003). Sens du travail. Définition, mesure et validation. Dimensions individuelles et sociales de l’investissement professionnel.

[CR29] Morin E (2006). Donner un sens au travail. Document—Centre de recherche et d’intervention pour le travail, l’efficacité organisationnelle et la santé (CRITEOS), HEC Montréal.

[CR30] Morin E (2008). *Sens* du travail, santé mentale et engagement organisationnel.

[CR31] Morin E, Archambault M, Giroux H (2001). Projet Qualité de Vie au Travail. Rapport Final.

[CR32] Morin E, Cherré B (1999). Les cadres face au sens du travail. Revue Française de Gestion.

[CR33] Morin E, Cherré B, Lancry A, Lemoine C (2004). Réorganiser le travail et lui donner du sens. La personne et ses rapports au travail.

[CR34] Morse NC, Weiss RS (1955). The function and meaning of work and the job. American Sociological Review.

[CR35] MOW International Research team (1987). The meaning of working.

[CR36] Muthén B (1984). A general structural equation model with dichotomous, ordered categorical, and continuous latent variable indicators. Psychometrika.

[CR37] Muthén LK, Kaplan D (1985). A comparison of some methodologies for the factor analysis of non-normal Likert variables. British Journal of Mathematical and Statistical Psychology.

[CR38] Muthén LK, Muthén BO (2010). Mplus user’s guide.

[CR39] Osborne, J. W. & Fitzpatrick, D. C. (2012). Replication analysis in exploratory factor analysis: what it is and why it makes your analysis better. *Practical Assessment, Research & Evaluation*, *17*(15), 1–8. 10.7275/h0bd-4d11.

[CR40] Osgood CE, Tannenbaum PH (1955). The principle of congruity in the perception of attitude change. Psychological Review.

[CR41] Pignault A, Houssemand C (2016). Construction and initial validation of the Work Context Inventory. Journal of Vocational Behavior.

[CR42] Pratt MG, Ashforth BE, Cameron KS, Dutton JE, Quinn RE (2003). Fostering meaningfulness in working and at work. Positive organizational scholarship.

[CR43] Rasch G (1980). Probabilistic models for some intelligence and attainment tests.

[CR44] Roe RA, Ester P (1999). Values and Work: Empirical findings and theoretical perspective. Applied Psychology: An international review.

[CR45] Ros M, Schwartz SH, Surkiss S (1999). Basic individual values, work values, and meaning of work. Applied Psychology: An international review.

[CR46] Rosso BD, Dekas KH, Wrzesniewski A (2010). On the meaning of work: A theoretical integration and review. Research in Organizational Behavior.

[CR47] Ruiz-Quintanilla SA, England GW (1994). How working is defined: Structure and stability.

[CR48] Siegrist JA (1996). Adverse health effects of high-effort/low-reward conditions. Journal of Occupational Health Psychology.

[CR49] Sommer KL, Baumeister RF, Stillman TF, Wong PTP (2012). The construction of meaning from life events: Empirical studies of personal narratives. The Human Quest for Meaning.

[CR50] Soper DS (2019). A-priori sample size calculator for structural equation models [Software].

[CR51] Steers RM, Porter L, Steers RM, Porter L (1979). Work and motivation: An evaluative summary. Motivation and work behaviour.

[CR52] Steger MF, Dik BJ, Duffy RD (2012). Measuring meaningful work: The work and meaning inventory (WAMI). Journal of Career Assessment.

[CR53] Streiner, D. L., Norman, G. R., & Cairney, J. (2015). Health measurement scales. *Oxford Medicine Online.*10.1093/med/9780199685219.001.0001.

[CR54] Super DE, Sverko B (1995). Life roles, values, and careers.

[CR55] Sverko B (1989). Origin of individual differences in importance attached to work: A model and a contribution to its evaluation. Journal of Vocational Behavior.

[CR56] Sverko B, Vizek-Vidovic V, Super DE, Sverko B (1995). Studies of the meaning of work: Approaches, models, models and some of the findings. Life roles, values, and Careers.

[CR57] Thurstone LL (1947). Multiple-factor analysis.

[CR58] Topalova V, Roe RA, Russinova V (1994). Changes in the attitude to work and unemployment during the period of social transition. Psychosocial aspects of employment: European perspectives.

[CR59] Valois P, Houssemand C, Germain S, Belkacem A (2011). An open source tool to verify the psychometric properties of an evaluation instrument. Procedia Social and Behavioral Sciences.

[CR60] Wanberg, C.R. (2012) The Individual Experience of Unemployment. *Annual Review of Psychology, 63* (1), 369-396. 10.1146/annurev-psych-120710-100500 10.1146/annurev-psych-120710-100500 21721936

[CR61] Warr P (2008). Work values: Some demographic and cultural correlates. Journal of Occupational and Organizational Psychology.

[CR62] Warr PB, Cook JD, Wall TD (1979). Scales of measurement of some work attitudes and aspects of psychological well-being. Journal of Occupational Psychology.

[CR63] Wrzesniewski A, Dutton JE, Debebe G (2003). Interpersonal sensemaking and the meaning of work. Research in Organizational Behavior.

[CR64] Wrzesniewski A, McCauley C, Rozin P, Schwartz B (1997). Jobs, careers, and callings: people’s relations to their work. Journal of Research in Personality.

[CR65] Yang C, Nay S, Hoyle RH (2010). Three approaches to using lengthy ordinal scales in structural equation models. Parceling, latent scoring, and shortening scales. Applied Psychological Measurement.

[CR66] Yu C-Y (2002). Evaluating cutoff criteria of model fit indices for latent variable models with binary and continuous outcomes. Dissertation.

[CR67] Zanders H, Ester P, Halman L, de Moor R (1993). Changing work values. The individualizing society. Value changes in Europe and North-America.

